# Semen Bacterial Concentrations and HIV-1 RNA Shedding Among HIV-1–Seropositive Kenyan Men

**DOI:** 10.1097/QAI.0000000000001244

**Published:** 2017-02-14

**Authors:** Christine J. Korhonen, Sujatha Srinivasan, Dandi Huang, Daisy L. Ko, Eduard J. Sanders, Norbert M. Peshu, John N. Krieger, Charles H. Muller, Robert W. Coombs, David N. Fredricks, Susan M. Graham

**Affiliations:** * Department of Epidemiology, University of Washington, Seattle, WA;; † Vaccine and Infectious Disease Division, Fred Hutchinson Cancer Research Center, WA;; ‡ School of Medicine, University of Washington, Seattle, WA;; § Nuffield Department of Medicine, University of Oxford, Oxford, United Kingdom;; ‖ Kenya Medical Research Institute-Wellcome Trust Research Programme, Kilifi, Kenya;; Departments of ¶ Urology;; # Medicine;; ** Laboratory Medicine;; †† Microbiology; and; ‡‡ Global Health, University of Washington, Seattle, WA; and; §§ University of Nairobi, Nairobi, Kenya.

**Keywords:** HIV-1, virus shedding, semen, RNA, viral, microbiota, viral load

## Abstract

**Introduction:**

HIV-1 is transmitted through semen from men to their sexual partners. Genital infections can increase HIV-1 RNA shedding in semen, but shedding also occurs in the absence of typical pathogens. We hypothesized that higher bacterial concentrations in semen would be associated with higher HIV-1 RNA levels.

**Methods:**

We analyzed semen samples from 42 HIV-1–seropositive Kenyan men using quantitative polymerase chain reaction (PCR) to assess bacterial concentrations and real-time PCR to measure HIV-1 RNA levels. Generalized estimation equations were used to evaluate associations between these 2 measures. Broad-range 16S rRNA gene PCR with pyrosequencing was performed on a subset of 13 samples to assess bacterial community composition.

**Results:**

Bacteria were detected in 96.6% of 88 samples by quantitative PCR. Semen bacterial concentration and HIV-1 RNA levels were correlated 0.30 (*P* = 0.01). The association between bacterial concentration and HIV-1 RNA detection was not significant after adjustment for antiretroviral therapy (ART) (adjusted odds ratio: 1.27, 95% CI: 0.84 to 1.91). Factors associated with semen bacterial concentration included insertive anal sex (adjusted beta 0.92, 95% CI: 0.12 to 1.73) and ART use (adjusted beta: −0.77, 95% CI: −1.50 to 0.04). Among 13 samples with pyrosequencing data, *Corynebacterium* spp., *Staphylococcus* spp., and *Streptococcus* spp. were most frequently detected.

**Conclusion:**

Most of these HIV-1–infected men had bacteria in their semen. ART use was associated with undetectable semen HIV-1 RNA and lower semen bacterial concentrations, whereas insertive anal sex was associated with higher bacterial concentrations. Additional studies evaluating the relationship between semen bacteria, inflammation, mucosal immunity, and HIV-1 shedding are needed to understand implications for HIV-1 transmission.

## Abstract 2

Supplemental Digital Content is Available in the Text.

## INTRODUCTION

HIV-1–infected men transmit HIV-1 through semen to their sexual partners.^1^ As the level of HIV-1 RNA in semen increases, the risk of transmitting HIV-1 increases.^2^ Urethritis has been demonstrated to increase semen HIV-1 RNA shedding, even among asymptomatic men and men taking antiretroviral therapy (ART).^3,4^ In the absence of classical sexually transmitted infections (STIs), HIV-positive men taking ART may still have detectible semen HIV-1 RNA.^5^ The semen microbiota contains a variety of bacteria in addition to classical STI pathogens^6–8^ including fastidious bacteria which can be identified and quantified using 16S rRNA gene polymerase chain reaction (PCR) assays.^9^ A better understanding of factors which increase HIV-1 RNA shedding in semen could result in interventions that reduce HIV-1 transmission from men to their sexual partners.

The goal of this study was to determine the association of semen bacterial concentrations with semen HIV-1 RNA levels in a cohort of HIV-1–positive Kenyan men. We hypothesized that higher semen bacterial concentrations would be associated with higher semen HIV-1 RNA levels. Secondary goals were to determine factors associated with higher semen bacterial concentrations in this population and to identify the bacterial species most commonly found in these samples.

## METHODS

### Study Population and Sample Collection

HIV-1–seropositive Kenyan men were recruited from a high-risk cohort including men with multiple sexual partners and men who had sex with men and enrolled between February 2005 and January 2008 into a prospective parent study assessing: (1) postprostatic massage fluid/urine compared with semen for evaluation of male genitourinary HIV-1 shedding,^10^ and (2) changes in seminal HIV-1 RNA shedding after ART initiation.^11^ Men were eligible if they were willing to attend up to 5 quarterly study visits; provide blood, urine, and semen samples; and observe 48 hours of sexual abstinence before each semen collection. At each study visit, participants were interviewed about their recent sexual history, number of male and female sexual partners, and health status. Subjects underwent a standardized examination including collection of urine and postprostatic massage fluid/urine to evaluate genital infections and inflammation.^10^ One week later, semen samples were collected either in the clinic or in participants' homes, with ejaculation into a sterile urine specimen cup or nonreactive condom supplied for this purpose (Durex Avanti; SSL International, Anderson, South California). Participants were asked to submit specimens within 2 hours of ejaculation. Samples with adequate semen quantity remaining after planned study analyses were included in this exploratory study.

### Ethics Statement

All participants provided written informed consent. This study was approved by the Kenya Medical Research Institute, the University of Washington, and the Fred Hutchinson Cancer Research Center.

### Laboratory Methods

Semen samples received by the laboratory were processed immediately after submission. After volume, appearance, and degree of liquefaction were noted, specimens were warmed in an incubator until liquefaction was complete. Sample aliquots were stored at −70°C until shipment to Seattle for further testing. Semen processing procedures were developed by a qualified andrologist (CHM), with quality assurance by the University of Washington Male Fertility Laboratory.

HIV-1 RNA was quantified in the University of Washington Retrovirology Laboratory. Vials were thawed and microcentrifuged at 16,000*g* for 15 minutes to separate seminal plasma and cell components. Seminal plasma aliquots (250 mL) were diluted 1:5 with Roswell Park Memorial Institute media,^12^ and centrifuged for 1 hour at 23,000*g*. Pellets were resuspended in bioMerieux lysis buffer and extracted using the MiniMAG Extraction system, which employs magnetic silica beads based on the Boom Technology (bioMerieux, Durham, NC).^13^ HIV-1 RNA was then quantified in seminal plasma using an independently validated TaqMan real-time RNA PCR (RT-PCR) amplification assay or the Amplicor HIV Monitor assay (Roche Molecular Systems, Pleasanton, CA).^13^ The lower limit for HIV-1 RNA detection was 150 copies per milliliter in plasma and in semen.

Blood CD4 cell counts were determined on-site using an automated method (FACS Count; Becton Dickinson, Forest Lakes, NJ). Additional screening included *Trichomonas vaginalis* culture (in-Pouch TV; BioMed Diagnostics, White City, OR), *Chlamydia trachomatis* and *Neisseria gonorrhoeae* testing (Aptima GC/CT Detection System; Hologic/GenProbe, San Diego, CA), and syphilis testing (rapid plasma reagin confirmed by *Treponema pallidum* hemagglutination assay).

Bacterial DNA was extracted from 100 μL of semen using the Bacteremia DNA Extraction Kit (Mobio, Carlsbad, CA). Bacterial concentrations were measured using broad-range quantitative PCR (qPCR) amplification of the 16S rRNA gene,^14^ and PCR inhibition was monitored using an amplification control.^15^ Species-specific qPCR assays were used to detect bacteria known to colonize the genitalia, including *Atopobium vaginae*, *Gardnerella vaginalis*, *Leptotrichia/Sneathia* spp., *Megasphaera* spp. types 1 and 2, bacterial vaginosis–associated bacterium (BVAB) 1 and 2, and *Mageeibacillus indolicus* (BVAB3).^16^

Broad-range 16S rRNA gene PCR targeting the V3–V4 region of the 16S rRNA gene was used to generate ∼470 bp amplicons, followed by pyrosequencing using the 454 Life Sciences Titanium technology (Roche, Branford, CT) to assess semen microbiota in a subset of samples. There were 13 samples from men not taking ART, with sufficient sample volume and bacterial DNA for this analysis.^14,17^ Six base-pair barcodes were used to multiplex the samples (see Supplemental Digital Content 1, http://links.lww.com/QAI/A950 for barcode sequences), and sequencing was performed from the V2 end of the 16S rRNA gene. Negative controls included sham digests which were processed in the same way as samples to assess contamination from DNA extraction or PCR reagents. We generated 38,159 reads with an average of ∼2900 reads/samples after filtering for length (minimum 250 bp), quality score (minimum 30), and removal of sequence reads originating from contaminants in PCR controls. Sequence reads were classified using the *pplacer* phylogenetic placement tool,^18^ with a curated reference set of urogenital bacteria.^14^ Query sequences were aligned and placed on a phylogenetic tree of reference sequences using *pplacer,* which finds the optimal insertion of reads on the tree according to maximum-likelihood or Bayesian posterior probability criteria. *pplacer* infers taxonomic classifications of sequence reads by associating edges of the phylogenetic tree with taxonomic labels. If a specific taxonomic rank is well represented in the phylogenetic tree, the query reads are classified at the species level. However, if there are novel sequences, or heterogeneity in the reads, the query reads are placed at higher taxonomic levels such as genus or family.^14^ Read sequences have been deposited to the NCBI Short Read Archive (SRP073630). Leukocytes were assessed by differential counting of cells in Wright–Giemsa–stained semen smears for the subset of samples undergoing broad-range PCR with pyrosequencing.

### Statistical Analyses

Only visits with data for both semen HIV-1 RNA and bacterial concentrations available were included for analysis. Chronic prostatitis score was determined using the National Institutes of Health Chronic Prostatitis Symptom Index.^19^ Missing laboratory results were carried forward from the last visit (7 CD4 counts) or imputed as negative when flanking visits were also negative (9 STI screening tests). Bacterial concentrations and HIV-1 RNA levels were log_10_ transformed.

Spearman rank correlation coefficient was used to test whether log_10_-transformed semen HIV-1 RNA or CD4 counts were associated with log_10_-transformed semen bacterial concentrations. Because over half the semen HIV-1 RNA levels were below the level of detection, semen HIV-1 RNA data were classified as undetectable if below the limit of detection (ie, <150 copies/mL). Generalized estimation equations (GEEs) with a logit link and exchangeable correlation matrix were used to assess associations between study variables and detectible semen HIV-1 RNA. GEEs with an identity link and exchangeable correlation matrix were used to assess associations between study variables and log_10_-transformed semen bacterial concentrations. Data were analyzed using Stata version 13.1 (StataCorp, College Station, TX).

For the analysis of semen bacterial concentration, using a minimum of 1 visit per subject, a sample size of 40 subjects would have 80% power to detect a mean difference in bacterial concentration of 1.3 log_10_ copies/100 μL between subjects with and without detectible HIV-1 RNA at the α = 0.05 level. With 2 visits per subject, using an exchangeable correlation matrix, this same sample size would have 80% power to detect a mean difference of 1.1 log_10_ copies/100 μL.^20^

## RESULTS

### Study Participants and Samples

Forty-two HIV-1–seropositive men enrolled between February 2005 and January 2008 had adequate semen samples for analysis (Table 1); this excludes 10 men from the original study with inadequate sample volumes. Men were similar to the original study population in participant characteristics (data not shown). The 42 participants contributed samples on 88 visits, with up to 3 visits per subject. Eleven of the 88 samples were collected off-site. For these samples, the average length of time from sample collection to receipt at laboratory was 52 minutes (range 18–120 minutes). Participants were taking ART during 34 visits (38.6%). Twenty-nine (85.3%) of 34 samples from men on ART had no detectable HIV-1 RNA.

**TABLE 1.**
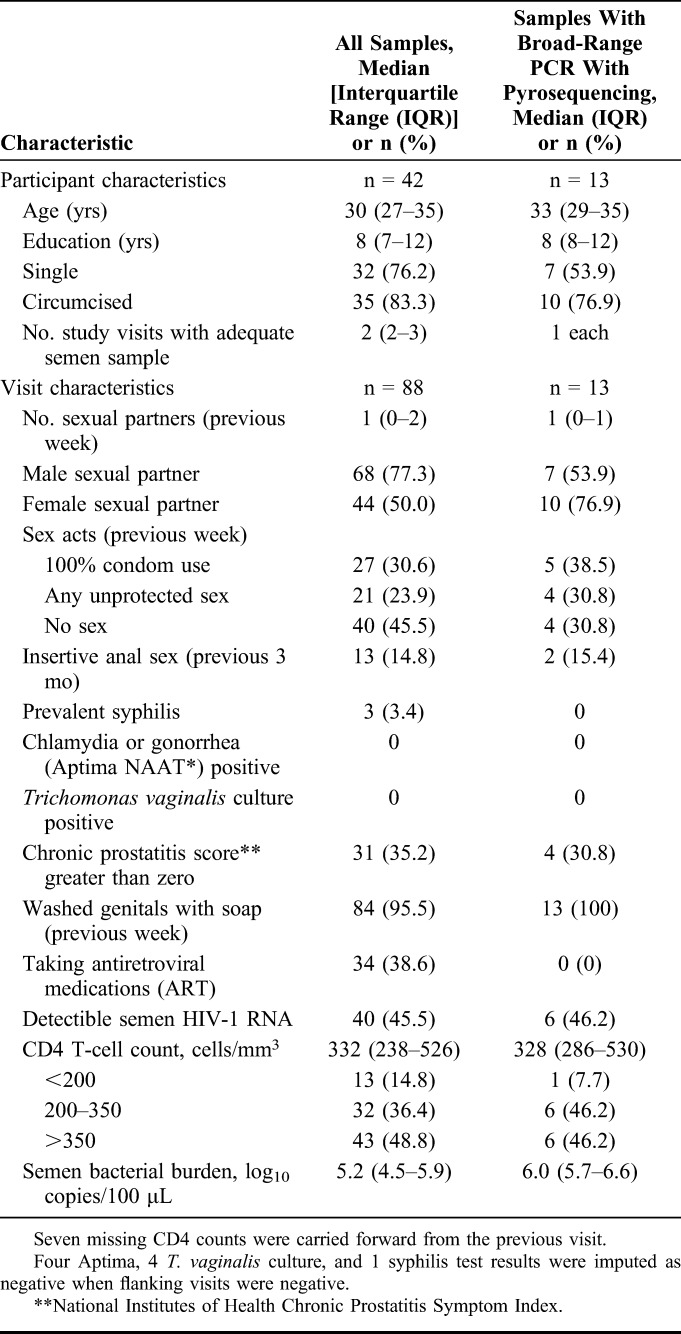
Participant and Visit Characteristics

### Semen Bacteria and HIV-1 RNA

**FIGURE 1.**
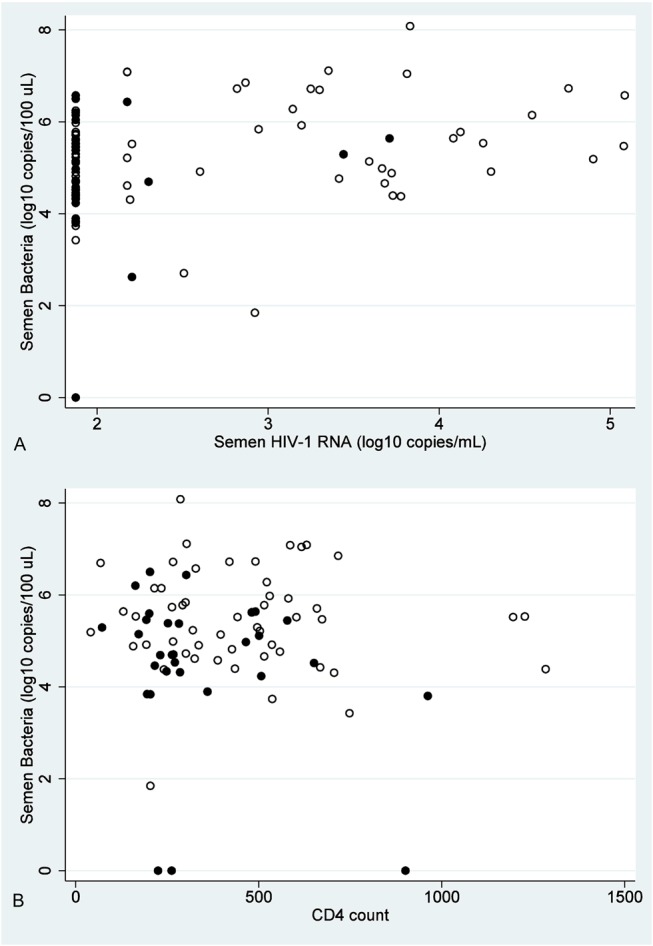
Semen bacterial concentrations and HIV-1 RNA or CD4 count. Panel A, Scatter plot of semen bacterial concentrations and semen HIV-1 RNA level; Panel B, scatter plot of semen bacterial concentrations and CD4 count. In each panel, black circles indicate visits when the participant was taking ART, whereas open circles indicate visits when no ART was used.

Bacteria were detected in 85 (96.6%) of 88 semen samples. Median bacterial concentrations were 5.2 log_10_ copies/100 μL (interquartile range, 4.5–5.9 log_10_ copies/100 μL). HIV-1 RNA was detected in 40 samples (45.5%). Among the 40 samples with detectable levels, the median HIV-1 RNA level was 3.4 log_10_ copies/mL (interquartile range, 2.5–3.8 log_10_ copies/mL). Spearman rank correlation coefficient for semen bacteria and HIV-1 RNA level was 0.30 (*P* = 0.01), whereas the rank correlation coefficient for semen bacteria and CD4 count was 0.01 (*P* = 0.90). Figure 1A presents semen bacterial concentrations and semen HIV-1 RNA levels. Figure 1B presents semen bacterial concentrations versus CD4 counts. In both figures, the ART status of the patient is indicated. When the sample was restricted to men not taking ART (N = 34 samples), the correlation coefficient for semen bacteria and HIV-1 RNA level was 0.22 (*P* = 0.11).

### Associations With Detectable Semen HIV-1 RNA

In bivariable analyses, semen bacterial concentration [odds ratio (OR) 1.49, 95% CI: 1.26 to 2.15] and ART (OR 0.09, 95% CI: 0.03 to 0.32) were both associated with detectible semen HIV-1 RNA. In the multivariable GEE model, only ART use was associated with detectible semen HIV-1 RNA (adjusted OR 0.11, 95% CI: 0.03 to 0.28, Table 2). In a model restricted to men not taking ART, no association was found between detectible semen HIV-1 RNA and semen bacterial concentration (OR 1.27, 95% CI: 0.78 to 2.08).

**TABLE 2.**
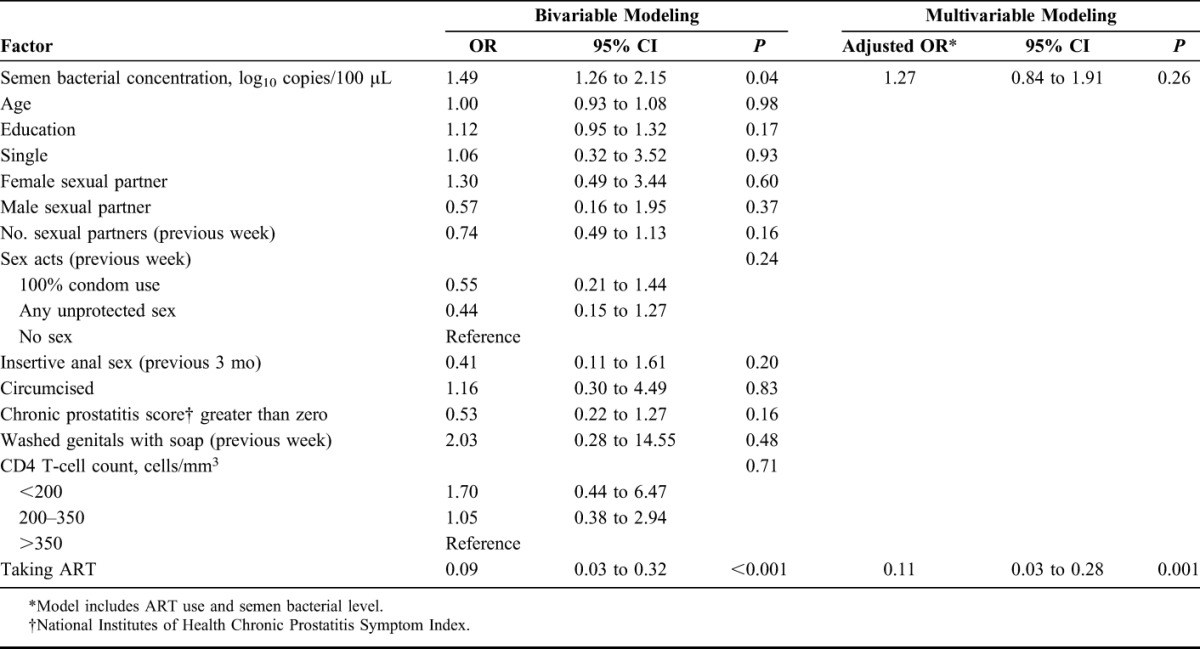
Factors Associated With Detectible Semen HIV-1 RNA

### Associations With Semen Bacterial Concentration

In bivariable analyses, detectible semen HIV-1 RNA was associated with higher semen bacterial concentration (beta 0.71 log_10_ copies/100 μL, 95% CI: 0.12 to 1.30 log_10_ copies/100 μL), whereas ART use was associated with lower bacterial concentration (beta −0.98 log_10_ copies/100 μL, 95% CI: −1.64 to −0.32 log_10_ copies/100 μL). In the multivariable GEE model, ART use was associated with lower semen bacterial concentration (adjusted beta −0.77 log_10_ copies/100 μL, 95% CI: −1.50 to −0.04 log_10_ copies/100 μL), and insertive anal sex was associated with higher semen bacterial concentration (adjusted beta 0.92 log_10_ copies/100 μL, 95% CI: 0.12 to 1.73 log_10_ copies/100 μL, Table 3).

**TABLE 3.**
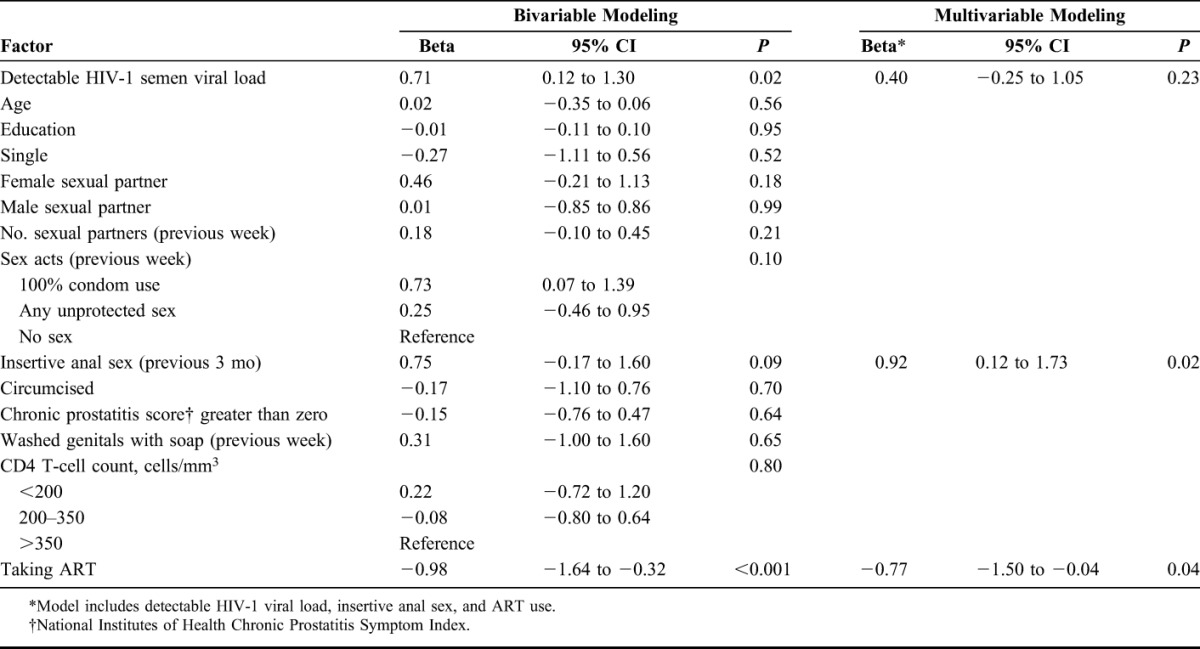
Factors Associated With Semen Bacterial Concentration (log10 copies/100 μL)

### Identification of Targeted Bacteria

Species-specific qPCR was performed on all 88 samples, and 17 (19.3%) were positive in at least 1 assay (Table 4). Among the 44 specimens from men who reported having female sexual partners in the period before sample collection, targeted bacteria were identified in 10 samples (22.7%). Among the 13 specimens from men reporting insertive anal sex, targeted bacteria were identified in 6 samples (46.2%). *Leptotrichia/Sneathia* spp. were the most commonly detected. *Leptotrichia/Sneathia* spp. were not associated with HIV-1 RNA shedding in this small sample (χ^2^ = 0.08, *P* = 0.8).

**TABLE 4.**
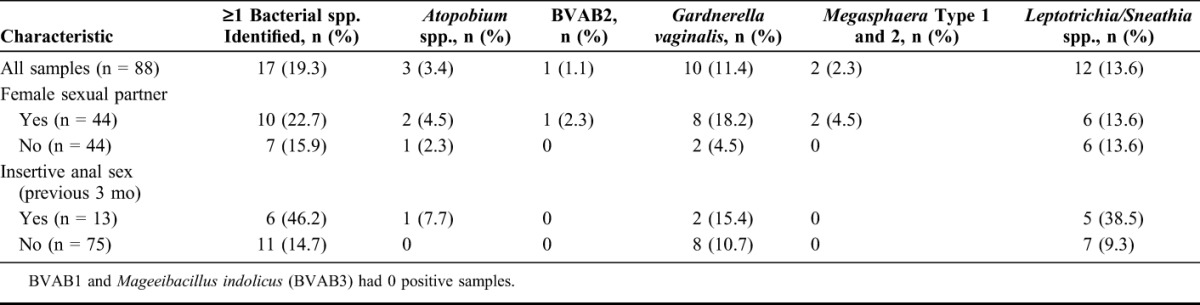
Bacterial Species Identified by qPCR in Semen Samples From 42 Subjects

### Bacterial Communities

Although bacteria were detected in 96.6% of semen samples using qPCR, targeted bacteria were detected in only 19.3% of samples. This suggests that the bacteria present were comprised mainly of species not specifically targeted using the taxon-directed qPCR assays. We applied broad-range PCR with pyrosequencing to a subset of samples from men not taking ART, with sufficient sample volume and bacterial DNA concentration (n = 13) for analysis. Six of the 13 had detectible HIV-1 RNA (Table 1).

**FIGURE 2.**
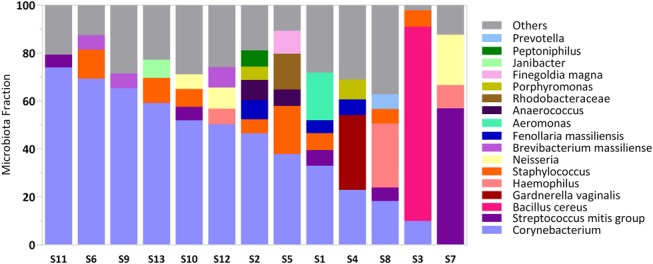
Bacterial communities in semen. Colored bars represent abundant bacterial taxa present at greater than 5% abundance in a single sample. All other taxa are grouped in the “other” category. Subjects S1 and S13 reported insertive anal intercourse. See Supplemental Digital Content 2, http://links.lww.com/QAI/A950 for complete data.

Broad-range PCR using primers targeting conserved regions of the 16S rRNA gene, with amplification of the intervening hypervariable region, does not require any a priori information about the bacterial community and can detect most bacteria. Using this approach, we found that all 13 samples contained *Corynebacterium* spp. ranging in relative abundance from 0.9% to 69%, with 6 samples having a bacterial community dominated by *Corynebacterium* spp. (≥50% relative abundance, Fig. 2. See Supplemental Digital Content 2, http://links.lww.com/QAI/A950 for complete listing). Other common skin bacteria, such as *Streptococcus* spp. and *Staphylococcus* spp., were detected in 12 and 13 samples, respectively, with 1 sample being dominated by streptococci (57% relative abundance). One sample was dominated by *Bacillus cereus* with a relative abundance of 81%. Vaginal bacteria were detected in ≥75% of samples and at a relative abundance ≥5% in at least 1 sample included *Prevotella*, *Porphyromonas*, and *Anaerococcus* spp. Other bacteria meeting the same metrics include *Brevibacterium massiliense*, *Fenollaria massiliensis*, and members of the *Rhodobacteraceae*. We detected *G. vaginalis* in only 3 samples with a maximum relative abundance of 31%. *Leptotrichia* species were detected in 5 samples, and *A. vaginae* in 2 samples at <2% relative abundance. BVAB1, BVAB2, *M. indolicus* (BVAB3), and *Megasphaera* spp. types 1 and 2 were not detected in any samples, consistent with the species-specific qPCR data. *Escherichia coli* sequences were detected in 6 samples by pyrosequencing at low relative abundance (0.02%–0.17% of sequence reads).

## DISCUSSION

The primary objective of this study was to evaluate the association between semen bacterial concentrations and semen HIV-1 RNA levels. Using bacterial 16S rRNA gene qPCR, we detected bacteria in most semen samples (96.6%). Overall, bacterial concentrations and semen HIV-1 RNA levels were correlated, but no association was found in the adjusted analysis. A recent study of the semen microbiome in HIV-infected men also reported correlation between semen bacterial concentrations and HIV-1 RNA levels before ART initiation, and hypothesized that semen bacteria may increase inflammation, HIV shedding, and transmission.^8^

One secondary goal of this study was to determine factors associated with higher semen bacterial concentrations. Overall, men taking ART had lower bacterial concentrations than those not taking ART. Men who reported insertive anal intercourse had higher semen bacterial concentrations. To our knowledge, this is the first time this association has been reported. One earlier study did find that men who had engaged in significantly higher rates of unprotected intercourse as the insertive partner in the previous 3 months had higher semen viral loads relative to their plasma viral loads.^21^ It seems plausible that insertive anal sex introduces bacteria from the anal cavity into the male genitourinary tract and semen, which might lead to inflammation and increased semen HIV-1 RNA levels.

Semen specimens were tested for bacteria hypothesized to be associated with insertive vaginal or anal sex; these same bacteria are also associated with bacterial vaginosis, a condition affecting 40%–50% of women in sub-Saharan Africa.^22,23^ *Leptotrichia/Sneathia* spp. were detected in 13% of samples and were more common in samples from men reporting insertive anal sex. *Leptotrichia/Sneathia* spp. have been associated with nonchlamydial nongonococcal urethritis in men^24^ and can be found in the vagina^25^ and the anal cavity.^26^ *Leptotrichia/Sneathia* spp. have been associated with increased HIV-1 RNA shedding in women on ART.^27^ It seems plausible that insertive anal sex may introduce bacteria from the anal cavity into the male genitourinary tract, leading to inflammation and increased semen HIV-1 RNA levels. Future studies should investigate the specific bacteria present in semen among men who engage in insertive anal intercourse.

Broad-range PCR with deep sequencing was applied to a subset of samples with sufficient DNA available. The sequence data were processed using our bioinformatics pipeline, which can classify sequences to the species level, facilitating identification of bacteria not reported in other semen microbiota studies, such as *B. massiliense* (prevalence, 92%) and *F. massiliensis* (prevalence, 77%). It is unlikely that these sequences are contaminants, as we included buffer and water controls when performing PCR amplification and sequencing. *B. massiliense* was first isolated from human ankle discharge,^28^ and *F. massiliensis* is a recently described isolate from a human osteoarticular sample.^29^ The most common bacteria detected included skin bacteria, such as *Corynebacterium*, *Staphylococcus*, *Streptococcus*, and *Dermabacter* species, consistent with published studies of the semen microbiota using cultivation and molecular approaches.^6,8,30^ As in these studies, we detected bacteria typically present in the vagina, such as *G. vaginalis*, *Prevotella*, *Peptoniphilus*, *Porphyromonas*, and *Anaerococcus* species, among others.^6,8,30^ *Lactobacillus crispatus* and *Lactobacillus iners* were detected, but were minority members of the community. *Bacillus cereus*, a recognized human pathogen which has been noted in nongastrointestinal tract infections and food poisoning,^31^ was dominant in 1 sample. *Mycoplasma* spp. and *Ureaplasma* spp., found in up to 19% of men seeking fertility treatment,^8,32,33^ and up to 33% of men suspected of urogenital tract infection,^34^ were relatively uncommon in our samples. *Mycoplasma hominis* was detected in 2 men (15%), whereas *Ureaplasma* was not detected (Supplemental Digital Content, Table 2, http://links.lww.com/QAI/A950). Bacteria typically present in the gut, such as *E. coli*, *Faecalibacterium prausnitzii*, and *Eubacterium rectale* were minority members of the semen microbiota, suggesting that, in general, semen bacteria are more similar to bacteria on skin and in the vagina, rather than in the gut, an observation made in other studies.^8,30^

Overall, these data largely agree with other reports of the semen microbiota.^8^ However, our classification pipeline offers greater granularity of bacterial species present. A key limitation is the small number of samples with sequence data due to relatively low bacterial concentrations. Future studies with a greater number of samples and with higher quantities of semen for DNA extraction will allow greater understanding of the role of the semen microbiota in HIV-1 shedding and transmission.

There are several limitations to this study. Because of the small sample size, power to detect differences was limited. Sample collection followed a detailed protocol, but some skin bacteria found in samples may have resulted from contamination by hand or penile skin flora. Because of limited sample volume, we were unable to identify other potential causes of increased semen HIV-1 RNA levels, such as cytomegalovirus shedding.^35,36^ Finally, this study took place among HIV-positive Kenyan men and may not be generalizable to other populations or locations. Strengths of the study include the successful collection of hard-to-obtain semen samples. Samples were processed by experienced researchers according to established methods.^11^ Finally, detailed bacterial testing was performed using state-of-the-art molecular methods.^37^

We found in this study that ART use was associated with lower semen bacterial concentrations and undetectable semen HIV-1 RNA. Untreated men had higher bacterial concentrations than treated men, possibly because of immunodeficiency. Men reporting insertive anal intercourse had higher bacterial concentrations than those who did not report this behavior. Additional studies are needed to determine the relationship between semen bacteria, inflammation, mucosal immunity, and HIV-1 shedding to understand the implication for HIV-1 transmission.
